# Dermoscopy in Leprosy: A Clinical and Histopathological Correlation Study

**DOI:** 10.5826/dpc.1102a32

**Published:** 2021-04-12

**Authors:** Alpana Mohta, Suresh Kumar Jain, Aditi Agrawal, Ramesh Kumar Kushwaha, Pritee Sharma, Khushboo Sethia, Manish Jain

**Affiliations:** 1 Department of Dermatology, Venereology and Leprosy, Sardar Patel Medical College, Bikaner, India; 2 Department of Dermatology, Venereology and Leprosy, Government Medical College, Kota, India; 3 Department of Dermatology, Venereology and Leprosy, RML Medical College and Hospital, New Delhi, India

**Keywords:** leprosy, dermoscopy, histopathology, clofazimine-induced pigmentation

## Abstract

**Background:**

Leprosy, an insidious infectious granulomatous disease, is diagnosed traditionally through clinical examination coupled with skin smears and histopathology. It has myriad clinical presentations that pose diagnostic challenges. Lately, dermoscopy has emerged as a rapid, noninvasive diagnostic modality for many dermatoses.

**Objectives:**

We evaluated the dermoscopic findings of various manifestations of leprosy and correlated them with clinical and histopathological features.

**Methods:**

This prospective, cross-sectional study was conducted in our skin outpatient department for a period of 1 year. Patients newly diagnosed as having leprosy or those undergoing leprosy treatment for less than 6 months were included. The most representative lesion was dermoscopically evaluated and later biopsied.

**Results:**

We included 73 patients in the study. Results indicated an obvious correlation between dermoscopic findings and histopathology. We noted orangish yellow and white structureless areas, steadily throughout the spectrum, depicting dermal granuloma. Additionally, we observed focal vascular structures such as branching, linear, and crown vessels that result from the pressure of granuloma pushing the dilated vessels upwards. The relative absence of skin appendages aided in differentiating leprosy from other granulomatous disorders. Novel findings of our study were the detection of a branch-like pattern of clofazimine-induced pigmentation on dermoscopy and orange globules on onychoscopy. Other unique findings included violaceous structureless areas, characteristic large telangiectatic vessels, follicular plugging, star-shaped silvery-white scaling, and white globules in type 1 reaction; white shiny steaks were observed in patients with borderline lepromatous leprosy, and central white dots and keratotic plugs were observed in patients with histoid leprosy.

**Conclusions:**

Dermoscopy, as a noninvasive modality, could aid in the quick diagnosis of leprosy and should be used as a handy tool to complement other investigative tools for this disease.

## Introduction

Leprosy is an insidious infectious granulomatous disease caused by *Mycobacterium leprae* [1]. Traditionally, it is diagnosed through clinical examination coupled with the slit-skin smear (SSS) test. Confirmation of the diagnosis requires the use of invasive techniques such as cutaneous and nerve histopathology. According to the World Health Organization, the disease is characterized by the presence of either hypopigmented or reddish skin lesions, with definite loss of sensation or peripheral nerve involvement (definite thickening with loss of sensation) and a positive SSS test result for acid-fast bacilli [2]. The clinical presentations of leprosy are classified by the Ridley–Jopling classification, according to the clinical, histopathological, bacterial, and immunological status of patients [3]. Histoid and indeterminate leprosy are rare forms of leprosy [4,5].

Leprosy reactions are generally an acute immunological phenomenon superimposed on the otherwise chronic course of leprosy. Type 1 reaction is a delayed hypersensitivity reaction with increased inflammation in preexisting lesions and neural dysfunction [6]. Type 2 lepra reaction, or erythema nodosum leprosum, manifests as evanescent, tender, superficial and deep erythematous papules and nodules with systemic features [7]. Cutaneous manifestations in patients with leprosy are seen secondary to clofazimine use in the form of hyperpigmentation [8].

Owing to such myriad presentations, leprosy has been rightfully called “the greatest imitator” [9]. Dermoscopy has emerged as a novel noninvasive modality for the rapid diagnosis of clinically confusing dermatoses, particularly the granulomatous disorders [10,11]. We evaluated the dermoscopic findings of various manifestations of leprosy and correlated them with clinical and histopathological features. We explored various features such as scaling patterns, vascular patterns, abnormal pigmentation, and appendageal abnormalities during the dermoscopic evaluation.

## Patients and Methods

This prospective, cross-sectional, noninterventional study was initiated after due approval from the Ethics Board of Government Medical College, Kota, Rajasthan, India and was conducted in the skin outpatient department of Government Medical College, Kota, Rajasthan India between July 2018 and January 2020. Patients newly diagnosed as having leprosy or those undergoing leprosy treatment for less than 6 months were included in the study. Written informed consent was obtained from all the patients before their enrolment in the study. A detailed cutaneous and neurological examination was conducted, and patients were categorized based on the Ridley–Jopling classification according to clinical, histopathological, and SSS results.

The most characteristic lesions from each patient were clinically photographed and later evaluated under a dermoscope (DermLite DL3N). The noncontact polarized mode was preferably used for visualizing blanchable structures such as vessel patterns and scaling. For the evaluation of superficial structures such as hair follicles and glands, the polarized mode was coupled with nonpolarized contact dermoscopy. Additionally, the same lesion (on which dermoscopy was done) was biopsied later for histopathological examination (using hematoxylin and eosin staining) and reported by a pathologist.

## Results

We examined a total of 73 patients (41 males and 32 females). The Fitzpatrick skin phototypes of the patients were between IV and VI. The most common form of leprosy encountered in our study was borderline tuberculoid, which was noted in 29 patients. Other clinical types included tuberculoid leprosy (n = 4), borderline lepromatous leprosy (n = 19), lepromatous leprosy (n = 17), and histoid leprosy (n = 4). Type 1 lepra reaction was noted in 7 patients with borderline tuberculoid leprosy, whereas 9 patients with lepromatous leprosy exhibited type 2 lepra reactions.

**Figure 1:**
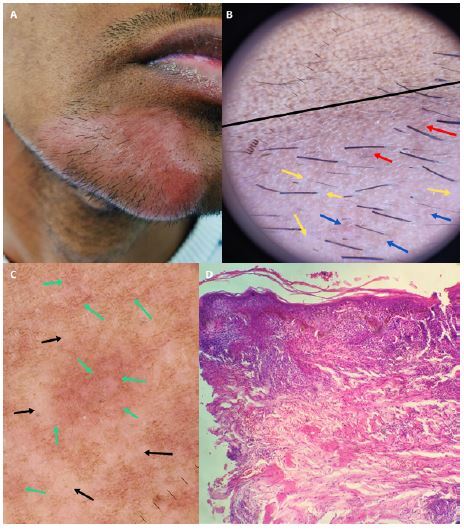
Tuberculoid leprosy. (A) Single, well-defined lesion with sparse hair. (B) On dermoscopy of the affected area (below the black line), orangish yellow structureless areas (blue arrows), white structureless areas (yellow arrow), peripheral erythema (red arrows), relative sparing of vellus hair, and extensive loss of pigment network (DermLite DL3, magnification ×10). (C) Telangiectatic vessels (green arrows), relative absence of hair follicles, loss of white dots (sweat gland openings), and extensive loss of the pigment network (black arrows) (DermLite DL3, magnification ×10). (D) Histopathology showed a well-formed dermal granuloma eroding the basal layer and involving the grenz zone and basal layer with damage to melanocytes (H&E, ×100).

Patients with tuberculoid leprosy (Figure 1) presented well-defined, annular, erythematous plaques in a saucer-shaped configuration with margins sloping towards the inside. On dermoscopic evaluation of the lesional edge, orangish yellow and white structureless areas were seen with peripheral erythema surmounted by telangiectatic vessels and moderate loss of hair follicles but relative sparing of vellus hair. Extensive loss of the pigment network with the absence of white dots was observed in a few cases. The histopathological finding was complementary to the dermoscopic finding, with well-formed dermal granulomas corresponding to white structureless areas (on dermoscopy) and dilated peripheral vessels corresponding to telangiectasia (Figure 1).

**Figure 2:**
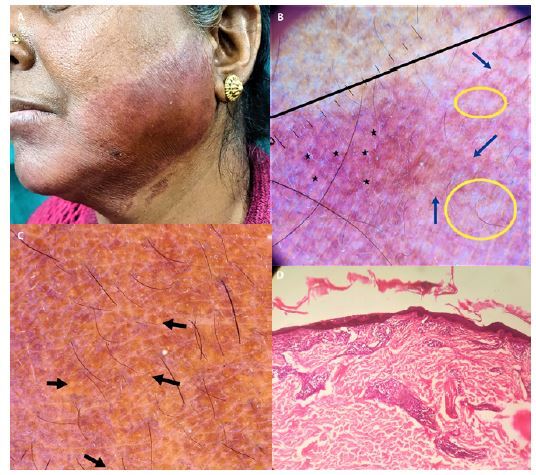
Borderline tuberculoid leprosy. (A) Single, well-defined lesion with pseudopodia and satellite lesions. (B) Dermoscopy of the lesion (below the black line) revealed yellowish orange structureless areas (yellow circles) surmounted by branching vessels (blue arrows), violaceous to erythematous hue in the background (black stars), patchy loss of the pigment network, diminished hair follicles and sweat glands (reduced white dots) (DermLite DL3, magnification ×10). (C) Presence of yellow dots and globules (black arrows) (DermLite DL3, magnification ×10). (D) Histopathological analysis revealed a well-formed, elongated dermal granuloma with a periappendageal granuloma involving the hair follicle (H&E, ×100) with sparing of the grenz zone.

**Figure 3:**
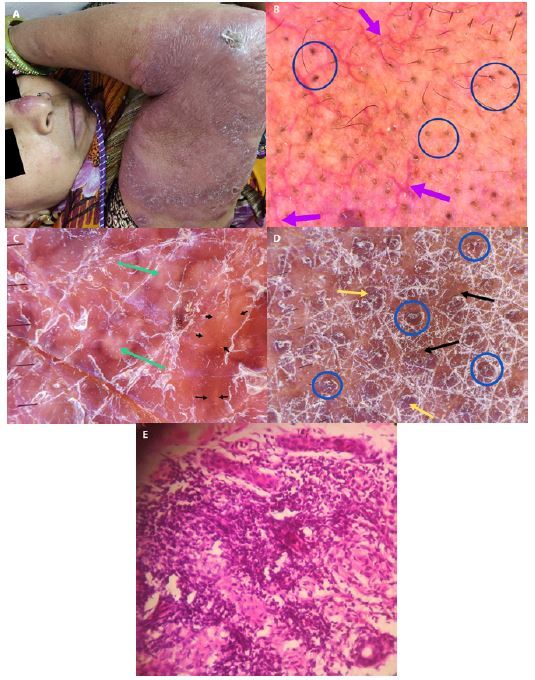
Borderline tuberculoid leprosy with type 1 lepra reaction. (A) Intense inflammation in preexisting plaques of borderline tuberculoid leprosy. (B) On dermoscopy, intense erythema with large telangiectatic vessels (unlike small branching vessels in borderline tuberculoid leprosy; purple arrows) and follicular plugging (blue circles). (C) White globules (green arrows), shiny scaling and accentuation of normal skin markings over features of borderline tuberculoid granuloma (orange-yellow structureless areas; small black arrows). (D) Follicular plugging (blue circles), intense violaceous to brown perifollicular and periappendageal (sweat gland) pigmentation (yellow and large black arrows), and triangular and star-shaped silvery white scaling (DermLite DL3, magnification ×10). (E) Histopathology showing acute inflammation around a dermal granuloma of borderline tuberculoid leprosy with dermal edema (H&E, ×400).

Clinical lesions in patients with borderline tuberculoid leprosy (Figure 2) displayed peripheral pseudopodia with satellite lesions. On dermoscopy, yellowish orange, structureless areas surmounted by branching vessels with patchy loss of the pigment network, diminished hair follicles, and sweat glands along with yellow dots and globules were observed. Regions of violaceous to erythematous hue were noted in the background (Figure 2). Patients with type 1 reaction exhibited intense erythema with large telangiectatic vessels (unlike small branching vessels in borderline tuberculoid leprosy), scaling, and follicular plugging. Additionally, we observed the accentuation of normal skin markings with triangular and star-shaped, silvery-white scaling and white globules. The histopathological analysis of borderline tuberculoid lesions displayed oval, elongated granulomas with intense inflammatory infiltration in type 1 reaction (Figure 3).

**Figure 4:**
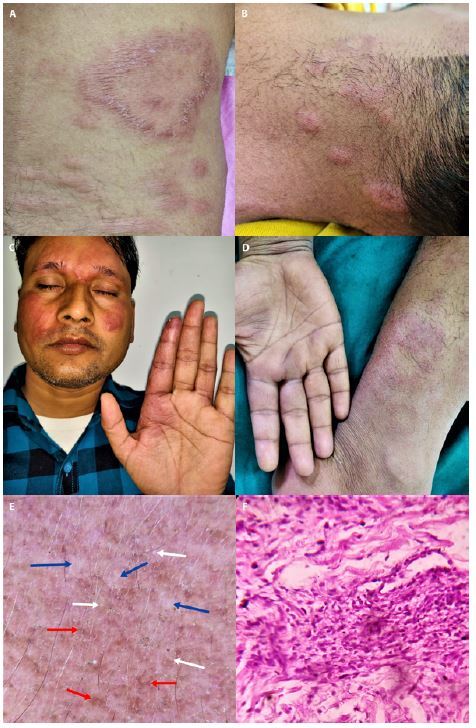
Borderline lepromatous leprosy. (A, B) Multiple erythematous or hypopigmented to skin-colored papulo-plaque lesions, with a punched-out appearance in some lesions. (C, D) Multiple well- to ill-defined erythematous plaques, involving the face and left palm (the relatively immune zone of sparing) of patient 1 and the palms and leg of patient 2, were a rare phenomenon. (E) Dermoscopic evaluation revealed small foci of loss of the pigment network (blue arrows) with focal areas of hyperpigmentation (red arrows) and relative sparing of appendages (presence of white dots) and hair follicles (DermLite DL3, magnification ×10). (F) On histopathological examination, loosely formed dermal granulomas, sparing of the grenz zone and presence of a few foamy macrophages (H&E, ×400).

Patients with borderline lepromatous lesions (Figure 4) presented loss of pigment networks with focal areas of hyperpigmentation. Other dermoscopic features included white, shiny streaks with relative sparing of appendages. No branching vessel was present. Histopathologically, loosely formed granulomas with foamy macrophages were present (Figure 4D), and SSS was positive for acid-fast bacilli. Detection of anesthetic lesions on the palms of 2 patients was an incidental finding of our study (Figure 4B).

**Figure 5:**
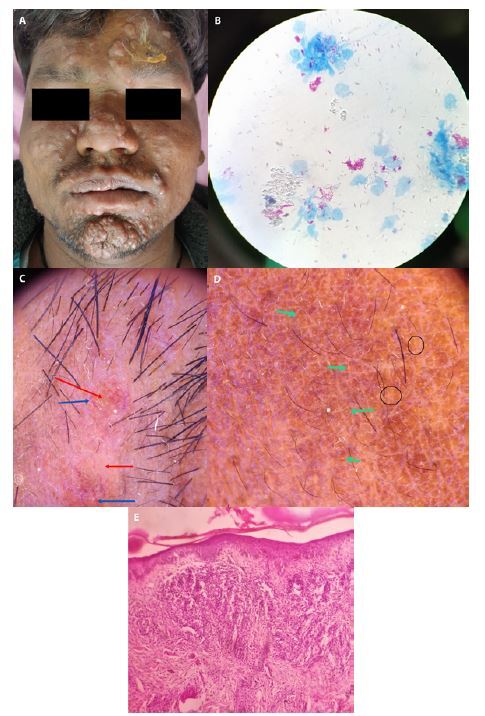
Lepromatous leprosy. (A) A case with multiple, symmetrical nodular lesions, depressed nasal bridge and supraciliary lateral madarosis. (B) Acid-fast staining of the SSS revealed a high bacteriological load with multiple acid-fast bacilli (Ziehl-Neelsen stain, ×1000). (C) Dermoscopy showed yellowish orange structureless areas (red arrow), shiny skin, telangiectatic vessels (blue arrows), and sparse appendages. (D) Sparing of the sweat glands (presence of white dots; green arrows), yellow globules (black circles), and accentuation of the normal reticular pigment network (DermLite DL3, magnification ×10). (E) Biopsy revealed diffusely distributed, ill-formed dermal granulomas, epidermal atrophy, and increased melanin in the basal layer (H&E, ×100).

Patients with lepromatous leprosy (Figure 5) exhibited variable presentations ranging from symmetrical macular to nodular and diffuse infiltrative forms. Yellowish orange and structureless areas with yellow globules were detected on dermoscopy. Accentuation of the normal reticular pigment network and xerosis were observed. Appendages were sparse. On histopathology, epidermal atrophy and ill-defined diffuse granulomatous infiltration with an abundance of foamy macrophages were observed. SSS was positive for acid-fast bacilli (Figure 5B).

**Figure 6:**
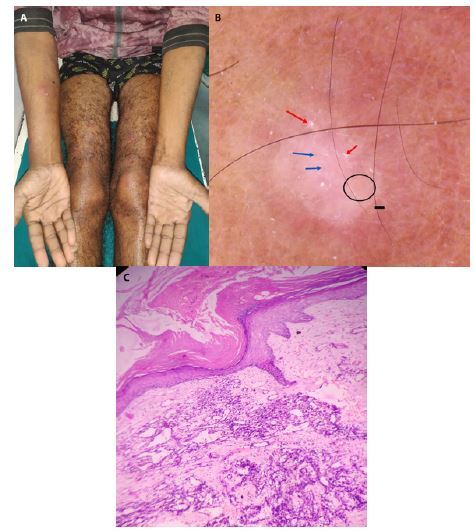
Erythema nodosum leprosum. (A) A case with multiple erythematous to violaceous nodules on the upper and lower limbs bilaterally. (B) Dermoscopic features of borderline lepromatous and lepromatous leprosy overlapping with increased erythema, shiny white scaling (red arrows), red dots (blue arrows), hypopigmented structureless areas (black arrow) and vascular dilatation (DermLite DL3, magnification ×10). (C) Marked vascularity and dermal edema with loosely formed granuloma (H&E, ×100).

In patients with erythema nodosum leprosum (Figure 6), the dermoscopic features of borderline lepromatous and lepromatous leprosy overlapped with increased erythema and vascular dilatation (Figure 6B). Similar features of borderline lepromatous and lepromatous leprosy with increased inflammation were observed on histopathology (Figure 6C).

**Figure 7:**
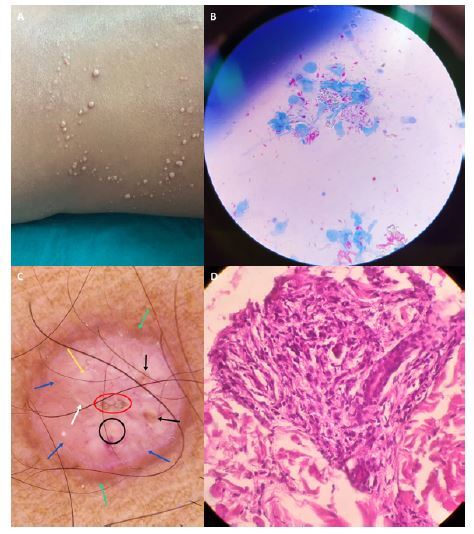
Histoid leprosy. (A) Multiple, flesh-colored, dome-shaped, shiny, succulent nodules over apparently normal skin with central umbilication, transepidermal elimination and signs of pseudokoebnerization in a few lesions. (B) Acid-fast staining of the SSS revealed a high bacteriological load with multiple, acid-fast bacilli (Ziehl-Neelsen stain, ×1000). (C) Dermoscopy showed crown vessels (blue arrows), central hypopigmented and blanchable domeshaped structures with perilesional hyperpigmentation (blue arrows), crystalline lines (white arrow), central white dots and keratotic plugs in central umbilication (red circle), yellowish orange structureless areas (black arrows), and shiny white areas (yellow arrow) (DermLite DL3, magnification ×10). (D) Expansile granuloma with spindle-shaped cells in the dermis (H&E, ×400).

Patients with histoid leprosy (Figure 7) displayed a unique clinical presentation of dermoscopic appearance, which included crown vessels with central hypopigmented and blanchable dome-shaped structures along with crystalline lines. Additionally, central umbilication displayed central white dots and keratotic plugs along with pseudokoebnerization. The presence of perilesional hyperpigmentation was also noted. Histopathology was also characteristically distinct in these patients (Figure 7D).

**Figure 8:**
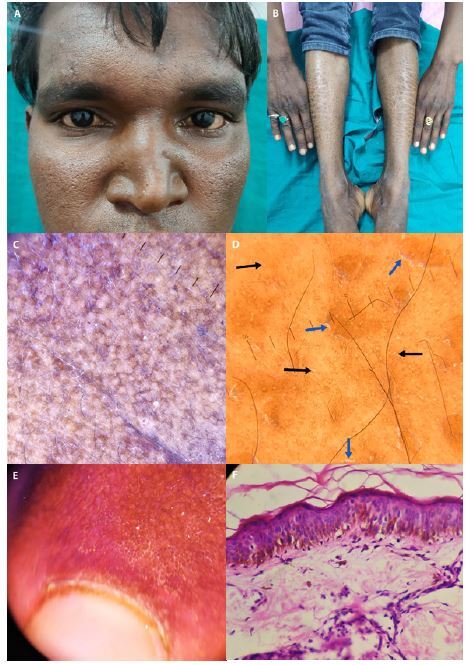
Clofazimine-induced pigmentation. (A) Clofazimine-induced orangish brown pigmentation of the skin and bulbar conjunctiva in a patient after 4 months of multibacillary multidrug therapy. (B) Clofazimine-induced ichthyosis on the distal limbs with orangish discoloration of the nails. (C) Dermoscopy revealed branching and honeycomb-shaped, brownish hyperpigmentation and the presence of white dots (sparing of sweat glands) (DermLite DL3, magnification ×10). (D) Clofazamine-induced ichthyosis with pavement-shaped structures, hexagonal zones of hyperpigmentation, loss of appendages, yellow dots (black arrows), and shiny scaling (blue arrows) (DermLite DL3, magnification ×10). (E) On onychoscopy, proximal nail fold branching and honeycomb-patterned pigmentation with interspersed orangish globules and shiny white scaling (DermLite DL3, magnification ×10). (F) Biopsy revealed a band of hyperpigmentation in the basal layer with increased melanin deposition and hyperkeratosis (H&E, ×400).

Clofazimine-induced pigmentation (Figure 8) was best visualized on dermoscopy as branching and honeycomb-shaped, brownish hyperpigmentation with yellow dots and globules. Similar nail fold pigmentation and orangish hue of the nail plate were observed in these patients. On onychoscopy, similar honeycomb pigmentation and interspersed orangish globules were seen in the proximal nail fold along with shiny white scaling, which was indicative of hyperkeratosis (Figure 8E). Other nonspecific changes included nail bed pallor and splinter hemorrhages. Clofazimine-induced ichthyosis on the distal limbs showed pavement-shaped structures with hexagonal zones of hyperpigmentation and loss of appendages. On histopathology, a band of hyperpigmentation was observed in the basal layer with increased melanin deposition (Figure 8F).

## Discussion

The multitudinous clinical presentations of leprosy have always posed a diagnostic dilemma to dermatologists. Often, the most challenging part is not the diagnosis of leprosy but the categorization of a patient’s clinical form, owing to such variable presentations. In many cases, categorization is possible only after the use of an invasive tool, such as histopathological analysis. However, dermoscopy has emerged as a safe, noninvasive modality for the diagnosis of countless dermatoses, including granulomatous disorders [10,11]. The dermoscopic characteristics of granulomatous disorders have been well established, and include yellowish orange structureless areas. These areas are better appreciated by blanching the vessels by applying pressure, and are formed due to the bulk of dermal granuloma and inflammatory infiltrates. Additionally, focal vascular structures such as branching, linear, and crown vessels are observed. These observations could be attributed to the pressure of granuloma that pushes the dilated vessels upwards [12–15].

In our study, these vascular structures were seen throughout the spectrum of leprosy in the patients. However, crown vessels were limited to the patients with histoid leprosy. These crown vessels are a variant of linear branching vessels that appear in the periphery, but towards the center they disappear abruptly without crossing the entire lesion [16]. Other common findings were dyspigmentation, appendageal loss, and dilatation.

Novel findings of our study are the detection of a branch-like pattern of clofazimine-induced pigmentation and the detection of orange-colored globules on onychoscopy. Clofazimine is said to extensively bioaccumulate and precipitate in almost all tissues, leading to the formation of crystal-like drug inclusions after around 4 months of administration as part of multibacillary multidrug therapy. However, studies have reported that partitioning of the freely circulating base form of the drug into the subcutis is the main cause of pigmentation [8].

Another unique finding of our study is the detection of violaceous structureless areas along with characteristic large telangiectatic vessels (unlike small branching vessels in borderline tuberculoid leprosy), follicular plugging, and star-shaped, silvery-white scaling and white globules in patients with type 1 reaction. This characteristic distribution of scaling contributes to epidermal hyperkeratosis, whereas the white globules occur secondary to dermal edema. We also observed the relative absence of skin appendages that we consider a specific finding and that can aid in differentiating leprosy from other granulomatous disorders. Another specific finding of this study is the detection of white shiny steaks in patients with borderline lepromatous leprosy that presumably occurred due to infundibular dilatation of hair follicles and hyperkeratosis.

We observed near total absence of white dots, which are actually the sweat gland openings around the tuberculoid pole, in patients with tuberculoid leprosy, borderline tuberculoid leprosy and type 1 reaction, with relative sparing of the same in patients with borderline lepromatous leprosy and lepromatous leprosy.

An incidental finding of our study is the detection of anesthetic lesions on the palms of 2 patients with borderline lepromatous leprosy. Although the palms and soles are relatively immune zones of sparing in leprosy, they may be involved in rare cases.

Similar studies have been conducted by Chopra et al [17] and Vinay et al [15] on 50 and 30 patients, respectively. Our study is the third study of this type that included a larger sample (73 patients) and the entire spectrum of leprosy including reactions, histoid leprosy, and clofazimine-induced skin changes. Vinay et al observed yellowish orange areas with vascular abnormalities consistently during the spectrum [15], and the finding is in close agreement with our findings. Similarly, we found decreased pigmentation in all lesions of leprosy without reaction, which is consistent with the observations of Chopra et al [17].

Dermoscopy is an upcoming diagnostic modality that holds leverage over other imaging techniques in being rapid and noninvasive [18]. Although many disease-specific signs have been delineated by dermoscopy, studies regarding the use of this modality in leprosy are meager. Our study could serve as a useful reference for studies on this subject in the future.

## Conclusions

In our study, the dermoscopic features of all forms of leprosy correlated well with histopathological findings. Although dermoscopy alone might not diagnose atypical lesions of leprosy, it could be a handy tool to complement SSS tests and histopathological findings in diagnosis. Some specific features such as yellowish orange structureless areas, vascular patterns, follicular plugging, and orange globules could help in diagnosing various forms of leprosy and reactions of clofazimine-induced pigmentation. However, the small sample size, exclusion of other granulomatous disorders, and exclusion of close clinical differential diagnoses of leprosy were limitations of our study.

## Figures and Tables

**Table 1:** Dermoscopic and Histopathological Findings in Leprosy Patients

Variant of Leprosy	Dermoscopic Features	Corresponding Histopathological Features
Tuberculoid leprosy	Orangish yellow and white structureless areas	Well-formed dermal granuloma eroding the basal layer
	Peripheral erythema Telangiectatic vessels	Vascular dilatation in the periphery of granuloma
	Moderate loss of hair follicles with relative sparing of vellus hair Absence of white dots (sweat gland openings)	Periappendageal granuloma leading to loss of hair follicles and sweat glands
	Extensive loss of pigment network	Involvement of grenz zone and basal layer with damage to melanocytes
Borderline tuberculoid leprosy	Yellowish orange structureless areas surmounted by branching vessels Violaceous to erythematous hue in background	Elongated dermal granuloma with vascular dilatation
	Patchy loss of pigment network Diminished hair follicles and sweat glands (reduced white dots)	Periappendageal granuloma
	Yellow dot and globules	Dilatation of pilosebaceous unit
Borderline lepromatous leprosy	Loss of pigment network with focal areas of hyperpigmentation	Dermal granuloma
	White shiny streaks with relative sparing of appendages	Perifollicular hyperkeratosis
Lepromatous leprosy	Yellowish orange structureless areas	Loosely formed diffuse dermal granuloma
	Shiny skin	Epidermal atrophy
	Sparse appendages and yellow globules	Periadnexal inflammation
	Accentuation of the normal reticular pigment network	Increased melanin in the basal layer
Type 1 lepra reaction	Intense erythema with large telangiectatic vessels (unlike small branching vessels in borderline tuberculoid leprosy) Intense violaceous to brown periappendageal pigmentation	Inflammation around dermal granuloma of borderline tuberculoid leprosy Vascular dilatation
	White globules	Dermal edema
	Scaling and follicular plugging Accentuation of normal skin markings with triangular and star-shaped silvery-white scaling	Hyperkeratosis
Type 2 lepra reaction	Dermatoscopic features of borderline lepromatous and lepromatous leprosy, overlapped with increased erythema and vascular dilatation Red dots	Marked vascularity with loosely formed granuloma
	Hypopigmented, structureless areas	Dermal edema
Histoid leprosy	Yellowish orange structureless areas Crown vessels Central hypopigmented and blanchable dome-shaped structures Perilesional hyperpigmentation	Expansile granuloma with spindle-shaped cells Upward displacement of dilated vessels by dermal granuloma
	Crystalline lines Shiny white areas	Dermal fibrosis
	Central white dots and keratotic plugs in central umbilication pseudokoebnerization	Transepidermal elimination
Clofazimine-induced pigmentation	Branching and honeycomb-shaped brownish hyperpigmentation especially around sweat gland openings and hair follicles Yellow dots and globules Presence of white dots (sparing of sweat glands)	Band of hyperpigmentation in basal layer with increased melanin deposition Pigment-laden macrophages
	Clofazamine-induced ichthyosis with pavement-shaped structures and hexagonal zones of hyperpigmentation Loss of appendages	Hyperkeratosis
	Onychoscopy: branching and honeycomb-patterned pigmentation Interspersed orangish globules in proximal nail fold Shiny white scaling	
